# Efficacy and Safety of Combined Androgen Deprivation Therapy (ADT) and Docetaxel Compared with ADT Alone for Metastatic Hormone-Naive Prostate Cancer: A Systematic Review and Meta-Analysis

**DOI:** 10.1371/journal.pone.0157660

**Published:** 2016-06-16

**Authors:** Tobias Engel Ayer Botrel, Otávio Clark, Antônio Carlos Lima Pompeo, Francisco Flávio Horta Bretas, Marcus Vinicius Sadi, Ubirajara Ferreira, Rodolfo Borges dos Reis

**Affiliations:** 1 Evidencias - A Kantar Health Company, Campinas, São Paulo, Brazil; 2 Comitê Brasileiro de Estudos em Uro-Oncologia - CoBEU, São Paulo, São Paulo, Brazil; Innsbruck Medical University, AUSTRIA

## Abstract

**Objective:**

Prostate cancer is the most common nonskin cancer and second most common cause of cancer mortality in older men in the United States (USA) and Western Europe. Androgen-deprivation therapy alone (ADT) remains the first line of treatment in most cases, for metastatic disease. We performed a systematic review and meta-analysis of all randomized controlled trials (RCT) that compared the efficacy and adverse events profile of a chemohormonal therapy (ADT ± docetaxel) for metastatic hormone-naive prostate cancer (mHNPC).

**Methods:**

Several databases were searched, including MEDLINE, EMBASE, LILACS, and CENTRAL. The primary endpoint was overall survival. Data extracted from the studies were combined by using the hazard ratio (HR) or risk ratio (RR) with their corresponding 95% confidence intervals (95% CI).

**Results:**

The final analysis included 3 trials comprising 2,264 patients (mHNPC). Patients who received the chemohormonal therapy had a longer clinical progression-free survival interval (HR = 0.64; 95% CI: 0.55 to 0.75; p<0.00001), and no heterogeneity (Chi^2^ = 0.64; df = 1 [p = 0.42]; I^2^ = 0%). The biochemical progression-free survival (bPFS) also was higher in patients treated with ADT plus docetaxel (HR = 0.63; 95% CI: 0.57 to 0.69; p<0.00001), also with no heterogeneity noted (Chi^2^ = 0.48; df = 2 [p = 0.79]; I^2^ = 0%). Finally, the combination of ADT with docetaxel showed a superior overall survival (OS) compared with ADT alone (HR = 0.73; 95% CI: 0.64 to 0.84; p<0.0001), with moderate heterogeneity (Chi^2^ = 3.84; df = 2 [p = 0.15]; I^2^ = 48%). A random-effects model analysis was performed, and the results remained favorable to the use of ADT plus docetaxel (HR = 0.73; 95% CI: 0.60 to 0.89; p = 0.002). In the final combined analysis of the high-volume disease patients, the use of the combination therapy also favored an increased overall survival (HR = 0.67; 95% CI: 0.54 to 0.83; p = 0.0003). Regarding adverse events and severe toxicity (grade ≥3), the group receiving the combined therapy had higher rates of neutropenia, febrile neutropenia and fatigue.

**Conclusion:**

The combination of ADT with docetaxel improved the clinical progression-free survival, bPFS and OS of patients with mHNPC. A superior OS was seen especially for patients with metastatic and high-volume disease. This contemporary combination therapy may now be offered as a first-line treatment for selected patients.

## Background

Prostate cancer is the most common nonskin cancer in older men in the United Kingdom (UK), United States (USA) and Western Europe [1]. It is often cured when diagnosed in a localized stage, and is responsive to various treatments even when advanced or metastatic. Up to 40% of detected cases will eventually progress to a metastatic stage [2, 3]. In patients with locally advanced, recurrent or metastatic tumors, the goals of therapy are to prolong survival and the progression-free interval, while maintaining a good quality of life (QOL) [1].

Since the 1940s, the primary therapy for men with metastatic prostate cancer has been ADT alone, to suppress the production of testosterone, either with surgical or chemical castration [4, 5]. Chemotherapy is typically initiated only after the patient no longer responds to ADT alone, when the disease enters a "castration resistant” state [5, 6].

An initial RCT published in 2004 evaluated the use of a chemohormonal therapy (estramustine phosphate plus ADT) for newly diagnosed patients with metastatic prostate cancer, and showed a longer cPFS for the combined modality (p = 0.03), although there was no significant difference in the overall survival [7].

A combination of docetaxel, a semi-synthetic second-generation taxane, with prednisone, was the first treatment that could significantly improve overall survival in men with metastatic castration-resistant disease [8, 9]. Many questions have been raised since then, as to whether administering chemotherapy to men with metastatic hormone-naive prostate cancer (mHNPC), before symptomatic disease progression after starting ADT, could improve the overall survival and the quality of life of the patients [10].

Some early clinical studies have shown that the addition of docetaxel to ADT significantly increased the clinical progression-free survival of patients with mHNPC [11–15]. However, the results for the overall survival remained controversial. In two different RCTs published in 2015, no differences were observed in the first RCT for the overall survival between the groups studied [11–13], while in the other [14, 15] the overall survival was superior for the group with a combination of docetaxel plus ADT.

This systematic review aims to evaluate the effectiveness and safety of docetaxel associated with standard ADT in the treatment of patients with mHNPC.

## Methods

### Study selection criteria

#### Types of Studies

Randomized controlled clinical trials (RCTs) with parallel design that compared the association of ADT and chemotherapy (docetaxel), versus ADT alone.

#### Types of participants

Patients aged ≥18 years with cytological or histological diagnosis of mHNPC.

### Search strategy for identification of studies

A wide search of the main computerized databases of interest was conducted, including EMBASE, LILACS, MEDLINE, SCI, CENTRAL, The National Cancer Institute Clinical Trials service, and The Clinical Trials Register. In addition, the abstracts published in the proceedings of the American Society of Clinical Oncology (ASCO), American Association for Cancer Research (AACR), European Society for Medical Oncology (ESMO), Society of Urologic Oncology (SUO) and American Urological Association (AUA) were also searched.

For MEDLINE, we used the search strategy methodology for randomized controlled trials [16] recommended by the Cochrane Collaboration [17]. For EMBASE, we used adaptations of this same strategy [16], and for LILACS, we used the search strategy methodology reported by Castro et al [18]. We performed an additional search on the SCI database looking for papers that were cited on the included studies. We added the specific terms pertinent to this review to the overall search strategy methodology for each database.

The overall search strategy was: #1 "androgens"(Pharmacological Action) OR "androgens"(MeSH Terms) OR "androgen"(All Fields); #2 “Deprivation”(All Fields); #3 "therapy"(All Fields) OR "therapeutics"(MeSH Terms) OR "therapeutics"(All Fields); #4 "docetaxel"(Supplementary Concept) OR "docetaxel"(All Fields); #5 Clinical Trial (ptyp).

Searches of electronic databases combined the terms #1 AND #2 AND #3 AND #4 AND #5 and didn’t have language or date restrictions.

### Critical evaluation of the selected studies

Two of the researchers gauged the title and abstract of all of the references redeemed by the search strategies. Every reference with the least indication of fulfilling the inclusion criteria was listed as pre-selected. We retrieved the complete article of all pre-selected references. According to the earlier reported criteria, two different researchers determined which articles to include or exclude. The eliminated trials and the reason they were eliminated are listed in this article. As for the included trials, data was withdrawn from all of them.

Details regarding the main methodology characteristics empirically linked to bias [19] were extracted with the methodological validity of each selected trial assessed by two reviewers (T.E.A.B and O.C). There was thorough attention given to some items: the generation and concealment of the sequence of randomization, blinding, application of intention-to-treat analysis, sample size pre-definition, loss of follow-up description, adverse events reports, the source of sponsorship and lastly, if the trial was multicentric.

### Data Extraction

The data were extracted by two independent reviewers. In order to identify the study, the name of the first author and year of publication were used. When necessary, all data were gathered directly from the text or determined from the information available. The data of all trials were based on the intention-to-treat principle; therefore, they compared all patients who were designated to one treatment and those who were designated to the other arm.

The primary endpoint was overall survival (OS: defined as the time from randomization to death from any cause).

Secondary endpoints were:

- Time to clinical progression or death (clinical progression-free survival; cPFS: defined as the time until increasing symptoms of bone metastases, progression according to RECIST, or clinical deterioration due to cancer according to the investigator’s opinion);
- Time to PSA progression, clinical progression or death (biochemical progression-free survival; bPFS or time to failure-free survival). If data on were not available, data on time to castration-resistant prostate cancer (tCRPC) was assessed;
- The number of patients that presented adverse events (grade ≥3), both hematological (anemia, neutropenia, febrile neutropenia and thrombocytopenia) and non-hematological (nausea, vomiting, diarrhea, fatigue, stomatitis, neuropathy (sensory or motor) and thromboembolism events);
- The quality of life (QOL).

### Analysis and Presentation of Results

Data were analyzed using the Review Manager 5.1.2 statistical package (Cochrane Collaboration Software) [20]. Dichotomous clinical outcomes are reported as risk ratio (RR) and survival data as hazard ratio (HR) [21]. The corresponding 95% confidence interval (95% CI) was calculated, considering *P* values less than 5% (p<0.05). A statistic for measuring heterogeneity was calculated through I^2^ method (25% was considered low-level heterogeneity, 25–50% moderate-level heterogeneity and >50% high-level heterogeneity) [22, 23].

In order to estimate the sheer benefits of progression-free survival and overall survival, we calculated the meta-analytic survival curves as suggested by Parmar et al [21]. In accordance to the inverse-variance method [24], a pooled estimate of the HR was measured by a fixed effect model. Hence, for efficiency of adverse events an HR or RR >1 favors standard arm (control), whereas an HR or RR <1 favors docetaxel plus ADT treatment.

We carried out an additional analysis using the random-effects model described by DerSimonian and Laird [25], to see if there was statistical heterogeneity found in the meta-analysis. Consequently, this provided a more conservative analysis. We executed the funnel plot test described by Egger et al [26] to determine the possibility of any publication bias. When the pooled results were significant, the number of patients needed to treat (NNT or NNH) to cause or to prevent one event was calculated by pooling absolute risk differences in trials included in meta-analyses [27–29]. For all analyses, a forest plot was generated to display results.

In the efficacy assessment, a subgroup analysis was planned to evaluate the influence of docetaxel plus ADT in men with mHNPC and high-volume disease (defined by the presence of visceral metastases or four or more bone lesions with at least one beyond the vertebral bodies and pelvis).

## Results

**Fig 1:**
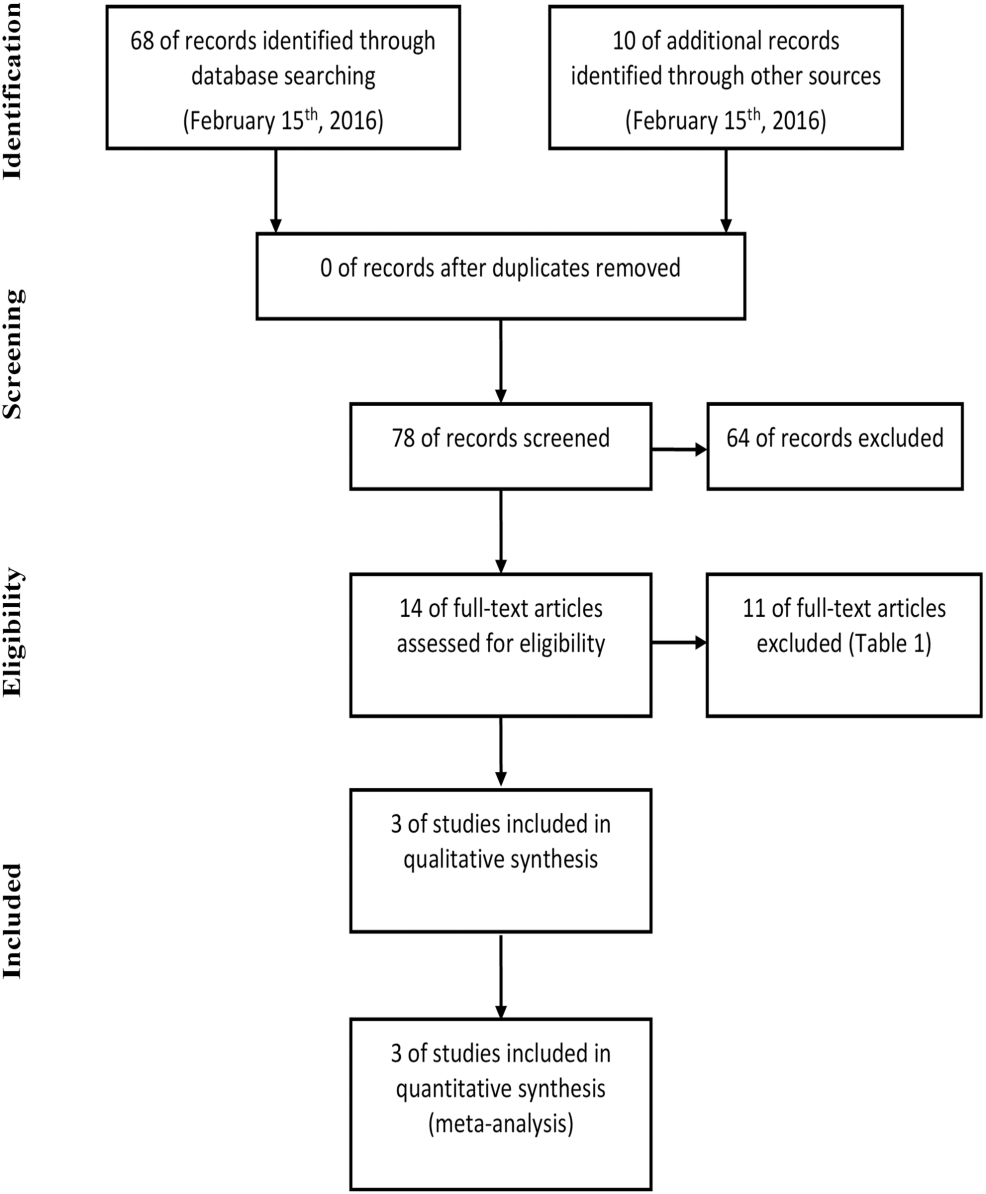
Trial selection flow.

The diagram represents the flow of identification and inclusion of trials, as recommended by the Preferred Reporting Items for Systematic reviews and Meta-Analyses (PRISMA) statement [30] (Fig 1 and S1 Table).

In the first search, 78 references were identified and screened. Fourteen were considered of potential interest and selected for analysis in full. Of these, 11 were excluded for different reasons and described in Table 1. The final analysis included 3 trials comprising 2,264 patients with mHNPC (Table 2).

**Table 1:** Characteristics of excluded studies.

Study	Reasons for exclusion
Fizazi 2015 [31]	High-risk localized prostate cancer
Fizazi 2015 [10]	Nonrandomized
Nakabayashi 2013 [32]	Nonrandomized
Thalgott 2014 [33]	Neoadjuvant treatment
Mottet 2012 [34]	Different comparison (ADT versus ADT plus radiotherapy)
Warde 2011[35]	Different comparison (ADT versus ADT plus radiotherapy)
Widmark 2009 [36]	Different comparison (ADT versus ADT plus radiotherapy)
Noguchi 2004 [7]	Different comparison (ADT versus ADT plus estramustine)
Millikan 2008	Different comparison (ADT versus ADT plus chemotherapy without docetaxel)
Amato 2013 [37]	Nonrandomized
Rajan 2015 [38]	Locally advanced or metastatic (without metastatic subgroup analysis)

ADT, androgen-deprivation therapy

**Table 2:** Characteristics of randomized studies evaluating ADT ± docetaxel for mHNPC.

Study	n	Type of study	Patients	Comparison	Primary endpoint	Median Follow-up (mo)
Gravis 2013/2015 [11–13] (GETUG-AFU 15 Trial)	385	Randomized, multicenter, phase III	mHNPC, ECOG PS 0–1, median age: 63 years	ADT	OS	83.9
				ADT + D		
Sweeney 2014/2015 [14, 15] (E3805: CHAARTED Trial)	790	Randomized, multicenter, phase III	mHNPC, ECOG PS 0–2, median age: 63 years	ADT	OS	29
				ADT + D		
James 2015 [39, 40] (STAMPEDE Trial) *	1,087	Randomized, multicenter, multi-arm	mHNPC, ECOG PS 0–2 median age: 65 years	ADT	OS	42
				ADT + D		
***Protocols***						
**Gravis 2013/2015** [11**–**13] **(GETUG-AFU 15 Trial)**						
ADT: LHRH receptor agonist or an LHRH, alone or combined with non-steroidal antiandrogens, or orchiectomy;						
ADT + D: 75mg/m^2^ of docetaxel every 3 weeks for a maximum of nine cycles.						
**Sweeney 2014/2015** [14, 15] **(CHAARTED Trial)**						
ADT: LHRH receptor agonist or an LHRH receptor antagonist or orchiectomy;						
ADT + D: docetaxel was given as 75 mg/m^2^ every 3 weeks for a maximum of six cycles.						
**James 2015** [39, 40] **(STAMPEDE Trial)**						
ADT: LHRH receptor agonist or an LHRH receptor antagonist or orchiectomy;						
ADT + D: docetaxel was given as 75 mg/m^2^ every 3 weeks for six cycles with prednisolone 10 mg daily.						

ADT, androgen-deprivation therapy; D, docetaxel, Mo, Months; ECOG; Eastern Cooperative Oncology Group; PS, performance status; OS, overall survival; mHNPC, metastatic hormone-naive prostate cancer.

* Only metastatic subgroup. For this analysis were excluded the results from other arms that employed different treatments

### Characteristics and results of included studies

#### GETUG-AFU 15 Trial [11–13] *(The French Genito-Urinary Tumor Group)*

This multicenter (29 in France and one in Belgium), open-label, phase III study analyzed patients with histologically confirmed adenocarcinoma of the prostate and radiologically proven metastatic disease and Karnofsky score of at least 70%. Patients were initially randomized 1:1 to receive ADT plus docetaxel (group 1) or ADT alone (group 2). ADT consisted of orchiectomy or luteinizing hormone-releasing hormone agonists, alone or combined with nonsteroidal androgen receptor inhibitors.

Randomization was done by a clinical research organization and was centralized nationally. Androgen deprivation therapy was given continuously until unacceptable toxic effects, or discontinuation at the patients’ request. In group 1, patients received 75 mg/m^2^ of docetaxel every 3 weeks for a maximum of nine cycles or it was discontinued prematurely in the case of progression, unacceptable toxic effects, or patients’ request. The primary endpoint was overall survival (Table 2).

In the ITT analysis, 385 patients (median age of 63 years) were randomized. Most patients had metastases at the time of diagnosis of prostate cancer (ADT: 75% and ADT + docetaxel: 67%); fifty-eight percent had a Gleason score of ≥ 8 (55% in group 1 and 59% in group 2). The median PSA level was ± 26 ng/ml.

After a median follow-up of 83.9 months, the bPFS was significantly longer for patients randomized to the ADT plus docetaxel arm for all patients, at 22.9 versus 12.9 months (HR: 0.67, 95% CI, 0.54–0.84). Similarly, cPFS was significantly longer for patients randomized to group 1 versus group 2, at 22.9 versus 15.3 months (HR: 0.69, 95% CI, 0.55–0.87). The OS also was increased with chemohormonal therapy compared with ADT alone, but the difference did not reach statistical significance (ADT plus docetaxel: median 62.1 versus 48.6 months for ADT alone, HR 0.88, 95% CI 0.68–1.14) (Table 3);

**Table 3:** Efficacy results of randomized studies evaluating ADT ± docetaxel for mHNPC.

Study	n (ITT)	Comparison	Median cPFS HR (95% CI)	Median bPFS or tCRPC HR (95% CI)	Median OS HR (95% CI)
Gravis 2013/2015 [11–13] (GETUG-AFU 15 Trial)	193	ADT	15.3 mo	12.9 mo	48.6 mo
	192	ADT + D	22.9 mo	22.9 mo	62.1 mo
			HR: 0.69 (0.55–0.87)	HR: 0.67 (0.54–0.84)	HR: 0.88 (0.68–1.14)
Sweeney 2014/2015 [14, 15] (E3805: CHAARTED Trial)	393	ADT	19.8 mo	11.7 mo	44.0 mo
	397	ADT + D	33.0 mo	20.2 mo	57.6 mo
			HR: 0.61 (0.50–0.75)	HR: 0.61 (0.51–0.72)	HR: 0.61 (0.47–0.80
James 2015 [39, 40] (STAMPEDE Trial)*	725	ADT	-	-	43 mo
	362	ADT + D	-	-	65 mo
				HR: 0.62 (0.54–0.71)	HR: 0.73 (0.59–0.89)

ADT, androgen-deprivation therapy; D, docetaxel, Mo, months; OS, overall survival; cPFS, clinical progression-free survival; bPFS, biochemical progression-free survival; tCRPC, time to castration-resistant prostate cancer; mHNPC, metastatic hormone-naive prostate cancer; ITT, intent to treat; HR: *hazard ratio*; CI, confidence interval.

*Only metastatic subgroup

In an unplanned subset analysis, there was no difference in OS for subgroups with low and high-volume disease. The cPFS was though better for the ADT + docetaxel therapy for the high volume disease subgroup (15.9 versus 9.7 months; HR: 0.61, 95% CI, 0.44–0.83).

In general, the most common grade ≥ 3 adverse events in the group 1 patients were neutropenia (0% versus 32%), febrile neutropenia (0% versus 7%) and fatigue (1% versus 7%). No serious adverse events were reported in the ADT alone group.

#### CHAARTED Trial [14, 15] (*E3805*: *Chemohormonal therapy versus androgen ablation randomized trial for extensive disease in prostate cancer*)

This multicenter, phase III study analyzed patients with histologically confirmed adenocarcinoma of the prostate and radiologically proven metastatic disease and ECOG PS of 0–2. Patients were initially randomized 1:1 to receive ADT plus docetaxel (group 1) or ADT alone (group 2). ADT consisted of orchiectomy or luteinizing hormone-releasing hormone agonists, or an LHRH receptor antagonist. In the group given ADT plus docetaxel, patients received 75 mg/m^2^ of docetaxel every 3 weeks for six cycles. The primary endpoint was overall survival (Table 2).

In the ITT analysis, 790 patients (median age of 63 years) were randomized. Most patients had metastases at the time of diagnosis of prostate cancer (73%), and they were never submitted to local treatment with intention to cure; sixty percent of the patients had a Gleason score of ≥8 (61.8% in group 1 and 60.7% in group 2), 97% were asymptomatic or oligosymptomatic (ECOG 0–1) and approximately 65% had a high-volume disease. The median PSA level was 50 ng/ml.

After a median follow-up of 29 months, the bPFS was significantly longer for patients randomized to group 1 for all patients, at 20.2 versus 11.7 months (HR: 0.61, 95% CI, 0.51–0.72). Similarly, cPFS was significantly longer for patients randomized to the ADT plus docetaxel arm at 33 versus 19.8 months (HR: 0.61, 95% CI, 0.50–0.75).

The OS also was increased with chemohormonal therapy compared with ADT alone (ADT plus docetaxel: median 57.6 versus 44 months, HR 0.61, 95% CI 0.47–0.80) (Table 3).

In a planned subset analysis, there was no difference in OS for the subgroup with low volume disease, while the OS was favorable in the high-volume disease subgroup for the ADT + docetaxel combination therapy (49.2 versus 32.2 months; HR: 0.60, 95% CI, 0.45–0.81) (Table 3).

In general, the most common grade ≥3 adverse events in group 1 patients were neutropenia (0% versus 12%), febrile neutropenia (0% versus 6%), and fatigue (0% versus 4.1%). Diarrhea, stomatitis, motor neuropathy, and sensory neuropathy occurred each at a rate of 1% or less.

The data related to the quality of life (QOL) were recently presented. Validated QOL instruments for prostate cancer and docetaxel including Functional Assessment of Cancer Therapy (FACT)–Prostate were administered at baseline and 3, 6, 9 and 12 months after randomization. Docetaxel is associated with decreased QOL on treatment (at 3 months) not seen with ADT alone. Nevertheless, in 12 months the QOL was better for the patients who had docetaxel versus ADT alone, returning to baseline. Moreover, this proposes that docetaxel + ADT does not confer a long-term negative impact on QOL for mHNPC [41].

#### *STAMPEDE trial* [39, 40] *(Systemic Therapy in Advancing or Metastatic Prostate Cancer*: *Evaluation of Drug Efficacy)*

This multicenter, multi-arm and multi-stage study analyzed 2,962 men with high-risk locally advanced or metastatic prostate cancer, all starting long-term ADT for the first time and ECOG PS of 0–2. As part of the protocol, a subset of 1.087 men with metastatic disease were randomly assigned to ADT plus docetaxel chemotherapy (group 1), or ADT alone (group 2). The ADT administered was similar to that of the CHAARTED study. Group 1 patients received 75 mg/m^2^ of docetaxel every 3 weeks for six cycles with prednisolone 10 mg daily. The primary endpoint was overall survival. The median PSA level was 65 ng/ml (Table 2).

After a median follow-up of 42 months, the OS was significantly increased in patients (M1) in group 1 compared to group 2 (65 versus 43 months, HR: 0.73, 95% CI, 0.59–0.89). A similar benefit was observed for bPFS (HR: 0.62, 95% CI, 0.54–0.71) (Table 3).

The study did not report data on adverse events for the subgroup of patients with metastatic disease. As a general rule, the introduction of docetaxel was associated with some additional toxicity compared with ADT alone, but the side effects were manageable, and few patients discontinued chemotherapy due to side effects.

### Meta-analyses

#### Efficacy

In the meta-analyses performed, the combination of docetaxel with ADT resulted in higher cPFS, bPFS and OS.

**Fig 2:**
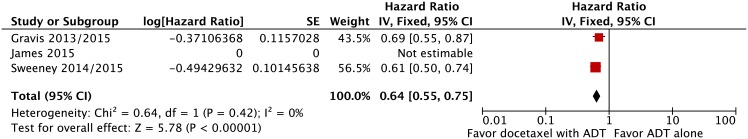
Comparative effect in clinical progression-free survival of ADT with docetaxel versus ADT alone. Abbreviations: ADT, androgen-deprivation therapy; CI, confidence interval.

The cPFS was clearly superior for patients treated with ADT plus docetaxel (HR = 0.64; 95% CI: 0.55 to 0.75; p<0.00001; NNT = 2), and no heterogeneity was found (Chi^2^ = 0.64; df = 1 [p = 0.42]; I^2^ = 0%) (Fig 2).

**Fig 3:**
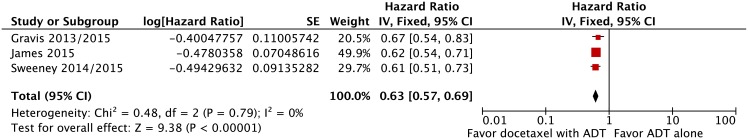
Comparative effect in biochemical progression-free survival of ADT with docetaxel versus ADT alone. Abbreviations: ADT, androgen-deprivation therapy; CI, confidence interval.

The bPFS was also significantly much better for patients treated with the chemohormonal regimen (HR = 0.63; 95% CI: 0.57 to 0.69; p<0.00001; NNT = 2). Also no heterogeneity was found (Chi^2^ = 0.48; df = 2 [p = 0.79]; I^2^ = 0%) (Fig 3).

**Fig 4:**
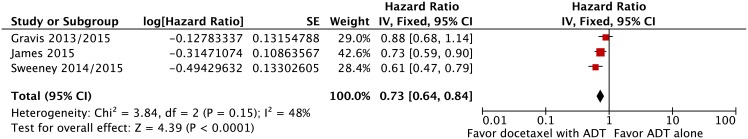
Comparative effect in overall survival of ADT with docetaxel versus ADT alone (Fixed-effect model analysis). Abbreviations: ADT, androgen-deprivation therapy; CI, confidence interval.

**Fig 5:**
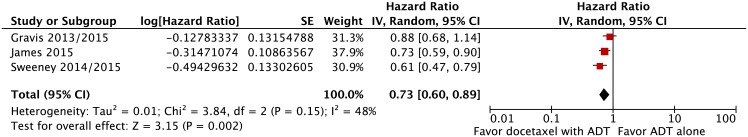
Comparative effect in overall survival of ADT with docetaxel versus ADT alone (random-effects model analysis). Abbreviations: ADT, androgen-deprivation therapy; CI, confidence interval.

The overall survival was also much longer for patients who received ADT plus docetaxel (HR = 0.73; 95% CI: 0.64 to 0.84; p<0.0001; NNT = 3) with moderate heterogeneity (Chi^2^ = 3.84; df = 2 [p = 0.15]; I^2^ = 48%) (Fig 4). We additionally performed a random-effects model analysis, in which the results remained favorable for the chemohormonal therapy (HR = 0.73; 95% CI: 0.60 to 0.89; p = 0.002) (Fig 5).

As an additional attempt to investigate the heterogeneity found in survival analyses, we excluded the GETUG-AFU 15 study [11–13], that although had a longer follow up, had also fewer patients, a lower median PSA value and the longest docetaxel exposure (9 cycles). After that, the overall survival still remained superior for the ADT plus docetaxel group (HR = 0.68; 95% CI: 0.58 to 0.80; p<0.00001), and no heterogeneity was found (Chi^2^ = 1.09; df = 1 [p = 0.30]; I^2^ = 9%).

#### Subgroup analysis

Two studies (GETUG-AFU 15 [11–13] and E3805: CHAARTED [14, 15]). The OS for low and high-volume diseases were reported separately.

**Fig 6:**
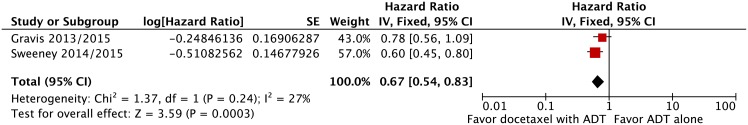
Comparative effect in overall survival of ADT with docetaxel versus ADT alone in patients with high-volume disease. Abbreviations: ADT, androgen-deprivation therapy; CI, confidence interval.

In the pooled analysis for high-volume disease patients, the OS was significantly longer for patients who received ADT plus docetaxel (HR = 0.67; 95% CI: 0.54 to 0.83; p = 0.0003; NNT = 3), with low-moderate heterogeneity (Chi^2^ = 1.37; df = 1 [p = 0.24]; I^2^ = 27%) (Fig 6). We also performed a random-effects model analysis, in which the results still remained favorable for the chemohormonal regimen (HR = 0.67; 95% CI: 0.52 to 0.87; p = 0.003).

**Fig 7:**
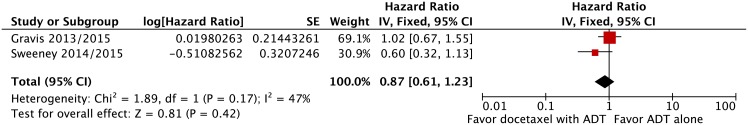
Comparative effect in overall survival of ADT with docetaxel versus ADT alone in patients with low-volume disease. Abbreviations: ADT, androgen-deprivation therapy; CI, confidence interval.

In the pooled analysis for low-volume disease patients, the OS was similar for patients who received ADT plus docetaxel and those with ADT alone (HR = 0.87; 95% CI: 0.61 to 1.23; p = 0.42), but with low-moderate heterogeneity (Chi^2^ = 1.89; df = 1 [p = 0.17]; I^2^ = 47%) (Fig 7).

#### Adverse events

**Fig 8:**
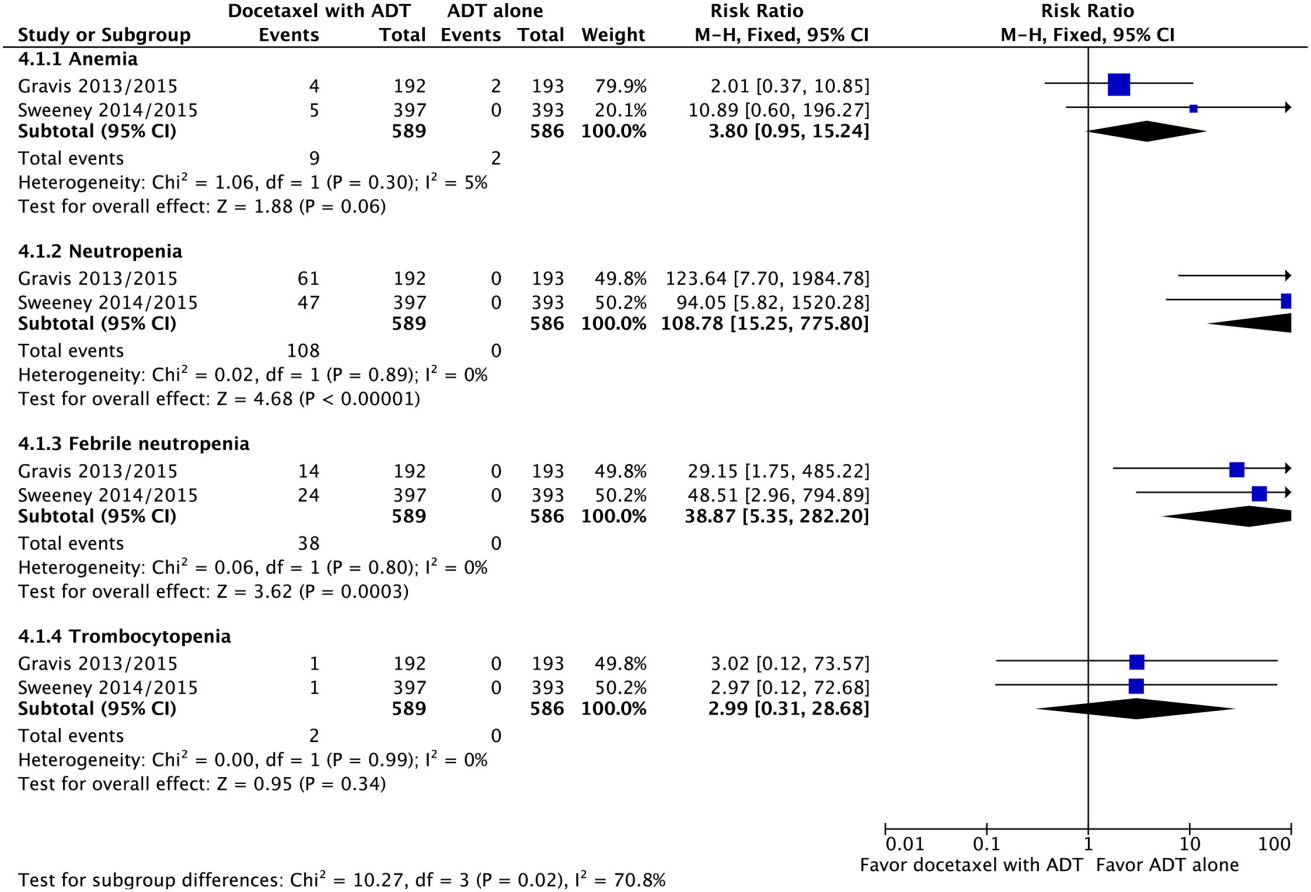
Comparative effect in hematologic toxicities of ADT with docetaxel versus ADT alone. Abbreviations: ADT, androgen-deprivation therapy; CI, confidence interval.

**Fig 9:**
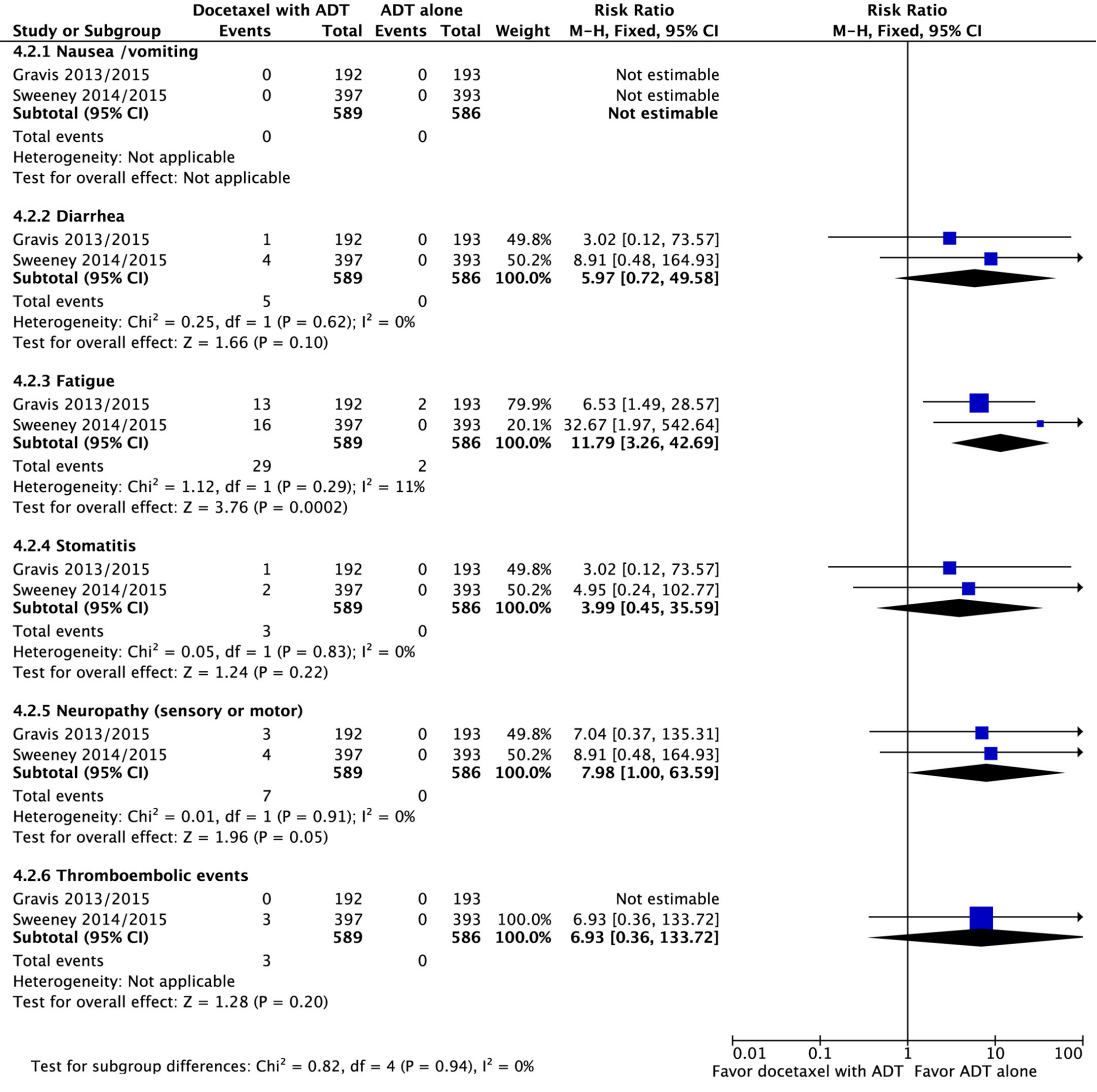
Comparative effect in non-hematologic toxicities of ADT with docetaxel versus ADT alone. Abbreviations: ADT, androgen-deprivation therapy; CI, confidence interval.

Regarding adverse events and severe toxicities (grade ≥3), the group receiving ADT plus docetaxel had higher rates of neutropenia (RR = 108.78 95% CI: 15.25 to 775.80; p<0.00001; NNH = 6), febrile neutropenia (RR = 38.87; 95% CI: 5.35 to 282.20; p = 0.0003; NNH = 17) and fatigue (RR = 11.79; 95% CI: 3.26 to 42.69; p = 0.0002; NNH = 20), without heterogeneity (Figs 8 and 9).

The probability of publication bias was low for all efficacy endpoints, according to the funnel plot analysis [26] (S1, S2 and S3 Figs; S2 Table).

## Discussion

Our review included the results of 3 studies [11–15, 39, 40] that evaluated the addition of docetaxel ± ADT for mHNPC. The meta-analysis has clearly demonstrated that the clinical progression-free survival, biochemical progression-free survival and overall survival were superior for patients who received the combination of docetaxel plus ADT.

Overall survival is considered the main outcome to be assessed for any cancer therapy, as it incorporates variables such as the mortality secondary to the natural history of the cancer itself, of the interventions used, and all the other intervening causes. Given the relatively indolent natural history of prostate cancer, it is anticipated that a longer follow-up is necessary to assess differences in overall survival [11–13, 42].

In this present meta-analysis, the overall survival was longer for patients who received the combination of chemotherapy plus ADT but with heterogeneity (I^2^ = 48%). As the random-effects models provides a more conservative estimate of the average treatment effect when trials are statistically heterogeneous [25], we performed a random-effects model analysis, in which results remained favorable to the use of ADT plus docetaxel (HR = 0.73; 95% CI: 0.60 to 0.89; p = 0.002).

We hypothesized about the heterogeneity found in the OS analysis. The presence of metastatic disease at the time of the diagnosis had just a little variation among the studies (61%-75%), as well as the ECOG PS. There was a difference in the time of follow-up of the studies (29 to 83.9 months), the median PSA value was lower in the GETUG-AFU 15 study [11–13], as well as it had the longest docetaxel exposure (9 cycles). When we excluded the study GETUG-AFU 15 [11–13] from the final OS analysis this study, the results still remained favorable for the chemohormonal regimen (HR = 0.68; 95% CI: 0.58 to 0.80; p<0.00001) and we found no heterogeneity (I^2^ = 9%).

Another very important issue that has limited the uniformity of the studies was the discrepancy found in the percentage of Gleason ≥8 patients included. As an example, in GETUG-AFU 15 Trial 55–59% were Gleason ≥8, as opposed to the CHAARTED Trial that included 60.7–61.8% of their patients with this high grade disease.

In the subgroup analysis, patients with high-volume disease in the GETUG-AFU 15 [11–13] did not show a significant difference in the primary end point of OS, had they received ADT plus docetaxel or ADT alone. In the E3805: CHAARTED study [14, 15] though, a subset analysis reported the greatest improvement in OS for this group of patients with high-volume disease. In the pooled analysis, the OS was higher for patients who received ADT plus docetaxel, with low-moderate heterogeneity (I^2^ = 27%). In the pooled analysis for the low-volume disease patients, the OS was similar for patients who received ADT plus docetaxel and those receiving ADT alone.

We hypothesized that maybe two main differences between these studies are causing this low-moderate heterogeneity: the number of patients with high-volume metastases in each study (GETUG-AFU 15: 52%; CHAARTED: 65%) and the number of docetaxel cycles used (GETUG-AFU 15: up to nine cycles; CHAARTED six cycles).

Although there may be some controversies regarding the definition of a high-volume disease in the mHNPC, at the St Gallen Advanced Prostate Cancer Consensus Conference (APCCC, 2015) [43], most of the panellists (61%) accepted the high-volume definition as used in CHAARTED [14, 15].

Another important issue to be considered is that the majority of patients (and not all of them) in this meta-analysis had metastatic disease before they started with the ADT. It is uncertain the real benefit of the chemohormonal treatment for patients who develop bony metastasis after the start of ADT only, or for those who develop biochemical recurrence after a definitive local treatment. Therefore, the results of this meta-analysis may not be extrapolated for these patients.

The three week interval regimen of docetaxel in combination with ADT used in all the three studies of this meta-analysis, was based on the two classic RCTs (SWOG 99–16 [8] e TAX 327 [9]) that for the first time showed a gain in OS for metastatic prostate cancer, when compared with mitoxantrone. More recently, another RCT with 346 patients compared docetaxel 75 mg/m^2^ IV on day 1 every 3 weeks given continuously, *versus* docetaxel 50 mg/m^2^ IV, on days 1 and 15, every 4 weeks [44]. Oral prednisolone 5 mg twice a day was administered for both groups. The primary endpoint was the time to treatment failure (TTTF). 170 patients in the 2-weekly group and 176 in the 3-weekly group were included in the analysis. The 2-weekly administration was associated with significantly longer TTTF than was 3-weekly administration (5.6 months, 95% CI 5.0–6.2 versus 4.9 months, 4.5–5.4; HR 1.3, 95% CI 1.1–1.6, p = 0.014). However, the PSA response rate and the OS were similar in both groups. Grade 3–4 adverse events occurred more frequently in the 3-weekly than in the 2-weekly administration group, including neutropenia (93 [53%] versus 61 [36%]), leucopenia (51 [29%] versus 22 [13%]), and febrile neutropenia (25 [14%] versus six [4%]). Neutropenic infections were reported more frequently in patients who received docetaxel every 3 weeks (43 [24%] versus 11 [6%], p = 0.002). The 2-weekly regimen was also associated with a better quality of life perception (p = 0.01) and less pain (p = 0.02) [45].

This present meta-analysis has demonstrated that grade 3–4 adverse events and toxicity, including neutropenia, febrile neutropenia and fatigue, occurred more frequently in the ADT plus docetaxel group. Only 1 study [41] reported QOL data comparing docetaxel + ADT versus ADT alone. However, it wasn’t possible to reach the pooled analysis of this endpoint in this revision. The data of this study suggests that docetaxel + ADT does not confer a long-term negative impact on QOL for mHNPC. We hypothesized that based on the above mentioned RCT [44], the 2-weekly docetaxel regimen may be a better option for association with ADT, when compared with the toxicity and grade 3–4 adverse events of the classic 3-weekly administration.

The National Comprehensive Cancer Network (NCCN) guidelines consider the combination of docetaxel plus ADT a valid option for patients with mHNPC [46]. The ESMO Clinical Practice Guidelines (2015) also recommended this scheme as a first-line treatment of mHNPC, in men fit enough for chemotherapy [6]. Half of the panellists of the St Gallen recommended the use of the docetaxel with ADT regimen in the majority of mHNPC patients with high-volume disease [43].

Lastly, docetaxel + ADT may be considered an option for Gleason 8–10 mHNPC patients, for patients with a poor PSA response to primary ADT, in cases with a rapid PSA doubling time, a disproportionately high or low PSA levels, bulky lymph node disease, and for extremely symptomatic patients. Additionally, men with a good PS, who are young or have little or no medical comorbidities, should also be considered for the combination of docetaxel and ADT therapy for increasing their time to disease progression and OS [47].

## Conclusion

The combination of docetaxel and ADT increased the clinical progression-free survival, bPFS and OS in patients with mHNPC (defined by the presence of visceral metastases or four or more bone lesions with at least one beyond the vertebral bodies and pelvis).The overall survival was especially higher for patients with a high-volume disease. This regimen may be offered as a first-line treatment for selected patients.

## Supporting Information

S1 Fig Funnel plot of clinical progression-free survival of ADT with docetaxel versus ADT alone. (PDF)

S2 Fig Funnel plot of biochemical progression-free survival of ADT with docetaxel versus ADT alone. (PDF)

S3 Fig Funnel plot of overall survival of ADT with docetaxel versus ADT alone. (PDF)

S1 Table Checklist of items to include when reporting a systematic review or meta-analysis. (PDF)

S2 Table Quality assessment (risk of bias) of randomized studies evaluating ADT ± docetaxel for mHNPC. (PDF)
