# Supplementary figures and images for: Efficacy and Safety of Combined Androgen Deprivation Therapy (ADT) and Docetaxel Compared with ADT Alone for Metastatic Hormone-Naive Prostate Cancer: A Systematic Review and Meta-Analysis
Source: PLoS One. 2016 Jun 16;11(6):e0157660. doi: 10.1371/journal.pone.0157660 (PMC4911003; doi:10.1371/journal.pone.0157660)

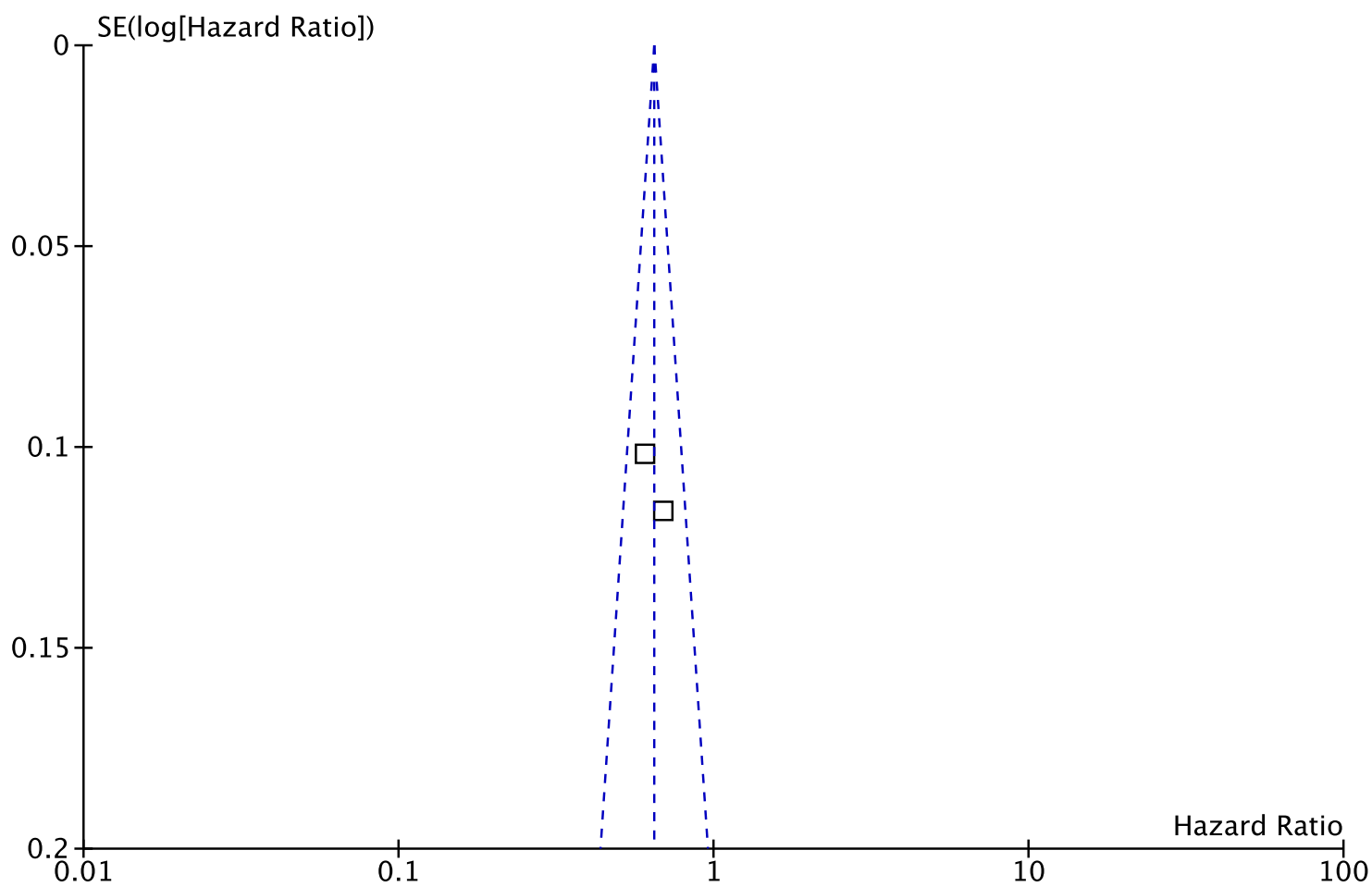

S1 Fig - Funnel plot of clinical progression-free survival of ADT with docetaxel versus ADT alone

Supplement: S1 Fig — (PDF) [file pone.0157660.s001.pdf]

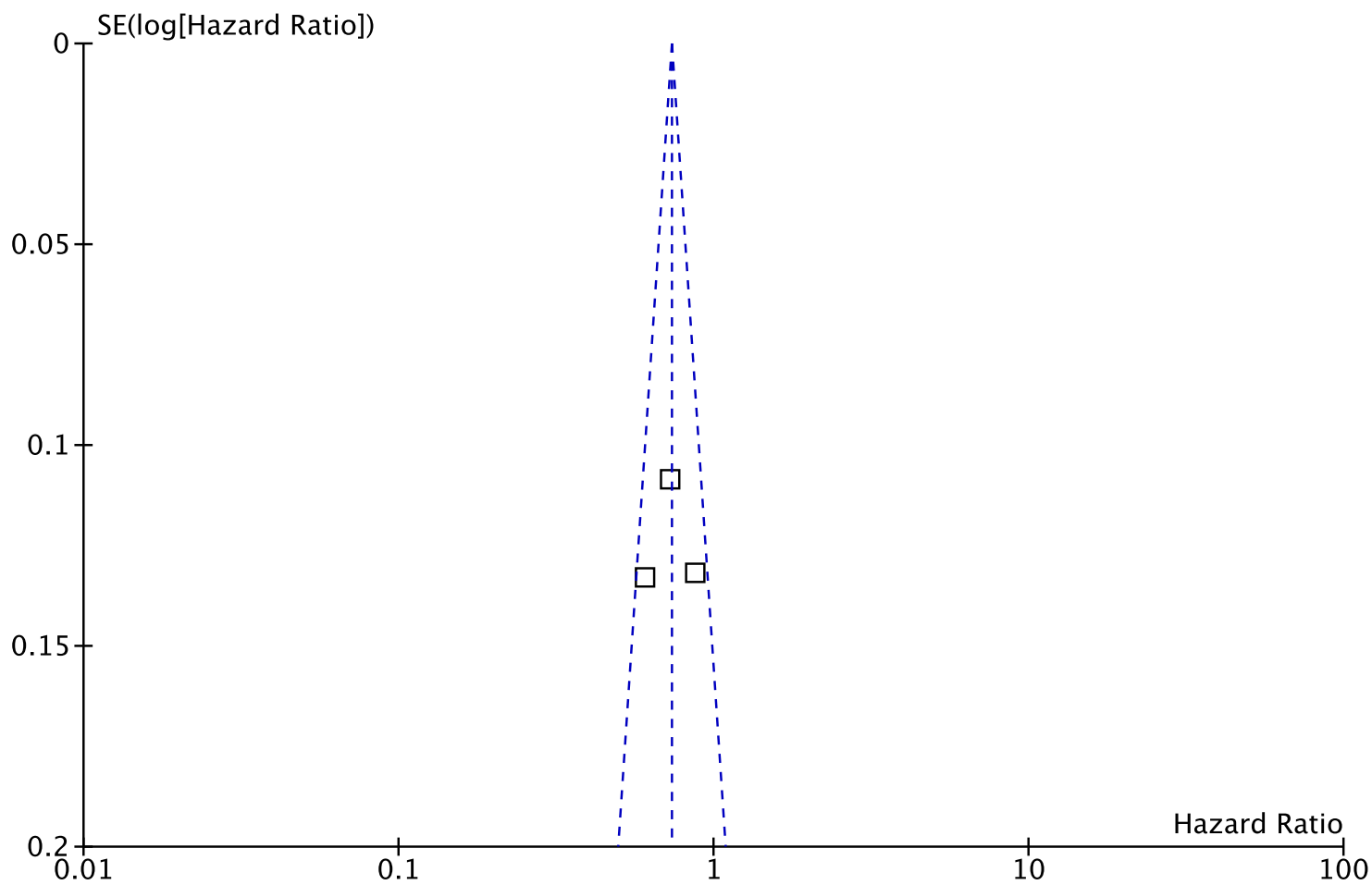

S3 Fig - Funnel plot of overall survival of ADT with docetaxel versus ADT alone

Supplement: S3 Fig — (PDF) [file pone.0157660.s003.pdf]
