# Supplementary material for: Efficacy and Safety of Combined Androgen Deprivation Therapy (ADT) and Docetaxel Compared with ADT Alone for Metastatic Hormone-Naive Prostate Cancer: A Systematic Review and Meta-Analysis
Source: PLoS One. 2016 Jun 16;11(6):e0157660. doi: 10.1371/journal.pone.0157660 (PMC4911003; doi:10.1371/journal.pone.0157660)
Supplement: S2 Fig — (PDF) [file pone.0157660.s002.pdf]

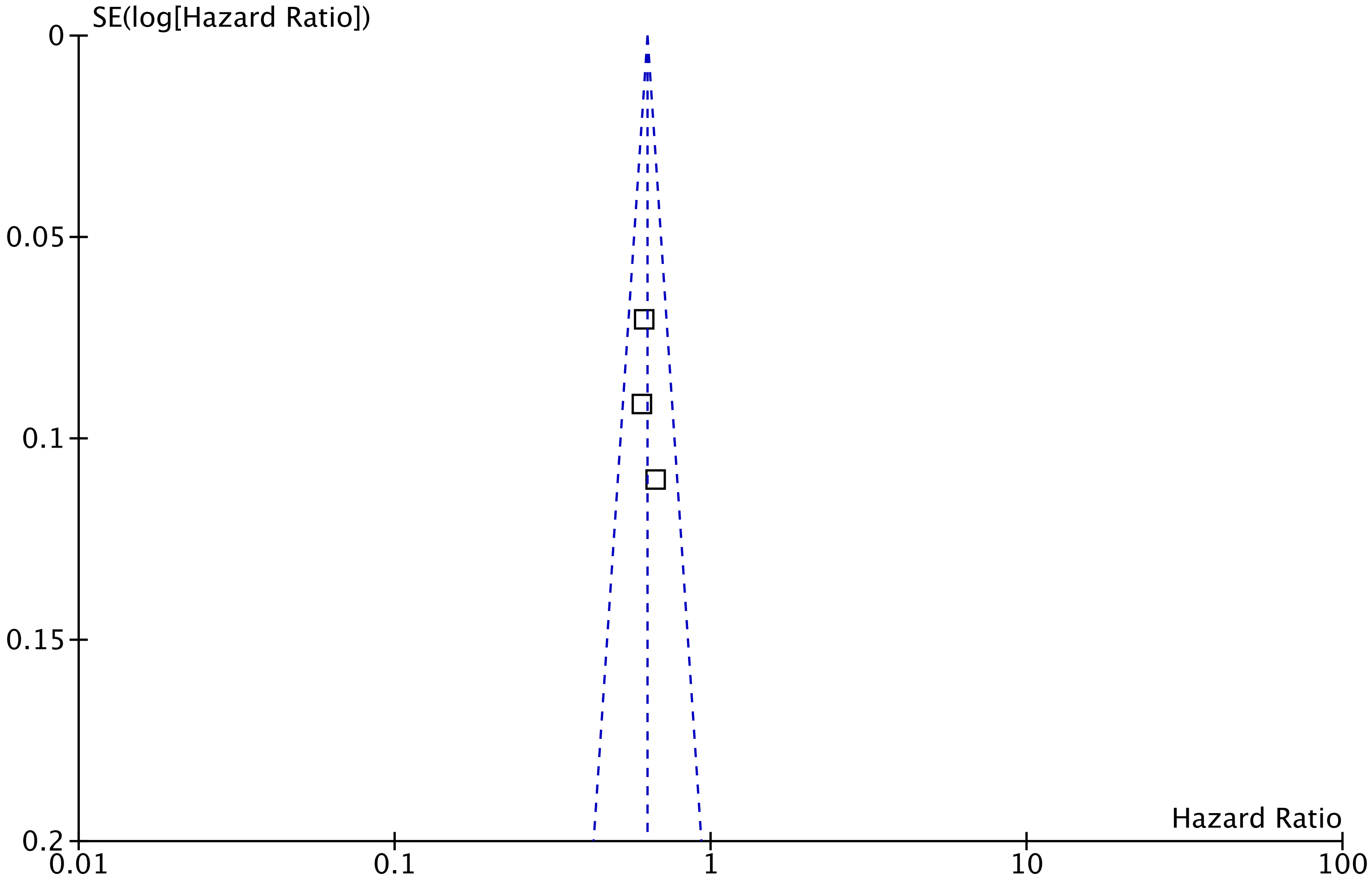

S2 Fig - Funnel plot of biochemical progression-free survival of ADT with docetaxel versus ADT alone
