# Supplementary material for: Efficacy and Safety of Combined Androgen Deprivation Therapy (ADT) and Docetaxel Compared with ADT Alone for Metastatic Hormone-Naive Prostate Cancer: A Systematic Review and Meta-Analysis
Source: PLoS One. 2016 Jun 16;11(6):e0157660. doi: 10.1371/journal.pone.0157660 (PMC4911003; doi:10.1371/journal.pone.0157660)
Supplement: S2 Table — (PDF) [file pone.0157660.s005.pdf]

S2 Table - Quality assessment (risk of bias) of randomized studies evaluating ADT ± docetaxel for mHNPC							
Study	Random sequence generation	Allocation concealment	Blinding of participants and personnel	Blinding of outcome assessment	Incomplete outcome data	Selective reporting	Other bias
Gravis 2013/2015 (11-13) (GETUG-AFU 15 Trial)	low risk	low risk	low risk	low risk	low risk	uncertain	uncertain
Sweeney 2014/2015 (14, 15) (E3805: CHAARTED Trial)	uncertain	uncertain	low risk	low risk	low risk	uncertain	uncertain
James 2015 (39, 40) (STAMPEDE Trial)	low risk	low risk	low risk	low risk	uncertain	uncertain	uncertain

Abbreviations: ADT, androgen-deprivation therapy; mHNPC, metastatic hormone-naive prostate cancer.
